# Nutrition and Frailty in the Older Adult: A Case-Based Approach to Assessment, Diagnosis, Intervention, Monitoring, and Evaluation in Preoperative Care

**DOI:** 10.3390/healthcare14213724

**Published:** 2026-11-03

**Authors:** Sandra Wolfe Citty, Amy Tyson, Sarah Mott, Laurence M. Solberg, Carmen Fernandez

**Affiliations:** 1 Department of Family and Community Health Systems, College of Nursing, University of Florida, Gainesville, FL 32610, USA; 2 Department of Geriatric Research Education and Clinical Center (GRECC), Malcom Randall Veterans Affairs Medical Center, Gainesville, FL 32608, USA; 3 Department of Surgical Services, North Florida South Georgia Veterans Health System, Gainesville, FL 32608, USA; 4 Department of Nutrition and Food Services, North Florida South Georgia Veterans Health System, Gainesville, FL 32608, USA; 5 Department of Internal Medicine, College of Medicine, University of Central Florida, Orlando, FL 32827, USA

**Keywords:** frailty, malnutrition, older adults, Mediterranean diet, prehabilitation

## Abstract

**Background/Objectives:** Frailty is a multidimensional syndrome characterized by reduced physiologic reserve and increased vulnerability to adverse outcomes, including postoperative complications, functional decline, and mortality. Malnutrition frequently coexists with frailty and contributes to muscle loss, impaired function, and decreased resilience. This narrative review explores the relationship between frailty and malnutrition and highlights practical approaches to assessment and management in older adults undergoing surgery. **Methods:** A narrative review of the current literature on frailty, malnutrition, comprehensive geriatric assessment, and prehabilitation was conducted. A fictional case study of Mrs. Jones, a 71-year-old woman undergoing evaluation for colon cancer surgery, was used to illustrate evidence-based assessment and management strategies. **Results:** Frailty and malnutrition interact in a reciprocal cycle that increases surgical risk and worsens health outcomes. Early screening can identify modifiable deficits, including weight loss, inadequate nutritional intake, impaired physical performance, functional limitations, and social barriers. Comprehensive geriatric assessment facilitates evaluation of medical, nutritional, functional, cognitive, and psychosocial factors. Evidence supports multimodal prehabilitation that combines individualized nutrition interventions, adequate protein and energy intake, exercise, medication optimization, oral-health management, and social support to improve surgical readiness and recovery. **Conclusions:** Routine screening for frailty and malnutrition, followed by comprehensive geriatric assessment and interdisciplinary management, can help identify reversible risk factors and guide individualized care. Integrating nutrition-focused interventions within prehabilitation programs may improve perioperative outcomes while supporting the preservation of strength, function, independence, and quality of life in older adults.

## 1. Introduction

Frailty is a clinical syndrome of increased vulnerability resulting from declines across multiple physiologic systems [1,2]. Frailty is often accompanied by sarcopenia, the loss of skeletal muscle mass and strength. Frailty is associated with falls, disability, hospitalization, institutionalization, reduced quality of life, and mortality [1]. Frailty is often recognized through physical manifestations such as weakness, reduced grip strength, slowed walking speed, fatigue, reduced activity, and unintentional weight loss; however, its origins and consequences extend beyond mobility alone [3]. An older person who is frail is at a significantly increased risk of morbidity and mortality, especially when faced with health stressors such as acute illness, chronic illness exacerbation (such as chronic pulmonary disease or congestive heart failure), or surgical procedures [4,5,6]. Nutrition is central to both the development and management of frailty [7].

Poor nutritional intake, inadequate protein and energy intake, micronutrient insufficiency, inflammation, and loss of lean body mass contribute to the progression of frailty [7]. Additionally, frailty may diminish an individual’s ability to shop for groceries, cook, eat independently, chew, or swallow, which can worsen nutritional risk [8]. In a study of older adults living in long-term care facilities, malnutrition was strongly associated with frailty; compared with normally nourished adults, malnourished adults had more than 10-fold higher odds of frailty [8]. For older adults, this relationship may be magnified by multimorbidity, polypharmacy, chronic pain, mobility limitation, social isolation, depression, poor oral health, food insecurity, and barriers to transportation or caregiving support [8]. Nutrition screening and assessment should be a core component of comprehensive frailty prevention and management in older adults [7].

In addition, older adults who require surgical procedures are at a significant risk of increased morbidity and mortality if they are frail before and after their surgical procedure [4]. Across recent studies, frailty consistently emerges as one of the strongest predictors of poor surgical outcomes in older adults. Frail patients experience significantly higher rates of postoperative complications, prolonged hospital stays, functional decline, and mortality [4]. Large multicenter studies demonstrate that frailty has an association with increased mortality risk in both elective and emergency surgery, even after adjusting for age and comorbidities, highlighting that frailty itself is a distinct and powerful risk factor [4,9]. A major strength of the Castillo et al. (2021) [4] study was its large, nationally representative sample of older adults undergoing emergency general surgery, which enhanced the reliability and clinical relevance of the findings. However, its retrospective design and reliance on a claims-based frailty index limited the ability to establish causation and may not have fully captured patients’ true clinical frailty [4]. The Hewitt et al. (2019) [9] study was strengthened by its prospective multicenter design, use of a validated frailty assessment tool, and inclusion of emergency surgical patients across all age groups. However, as an observational study conducted in selected UK hospitals, it may affect generalizability to other healthcare settings.

Frailty is also associated with higher odds of adverse outcomes, including postoperative delirium, infections, and failure to return to baseline independence [10]. More recent prospective studies demonstrate that frailty affects not only immediate postoperative outcomes but also long-term survival. Frail older adults are less resilient to surgical stress, more vulnerable to complications, and more likely to experience functional deterioration after surgery [11]. Together, these findings underscore the importance of frailty screening before and after surgical procedures, as early identification allows clinicians to optimize nutrition, mobility, and medical management to reduce risk and improve recovery trajectories [11]. This paper uses the case of Mrs. Sarah Jones, a 71-year-old female Air Force Veteran undergoing evaluation for colon cancer surgery, to illustrate a practical approach to the assessment and management of nutrition-related frailty in the preoperative setting.

## 2. Methods

This paper is a narrative review of frailty and its complex relationship with nutrition in older adults and presents a fictional case of an older adult who is undergoing an elective surgical procedure. For this narrative review, we searched PubMed/MEDLINE, CINAHL, and Google Scholar for English-language publications from January 2016 to September 2026. Landmark guidelines, consensus statements, and validation studies published before this window were included where they remain the primary source for a clinical definition or threshold. Search terms combined concepts related to frailty (e.g., “frailty,” “frail elderly,” “sarcopenia”), nutrition (e.g., “malnutrition,” “protein intake,” “Mediterranean diet,” “GLIM,” “nutrition screening”), and clinical application (e.g., “comprehensive geriatric assessment,” “polypharmacy,” “oral health,” “prehabilitation,” “Risk Analysis Index,” “Surgical Pause”). We prioritized clinical practice guidelines, consensus statements, systematic reviews and meta-analyses, and randomized controlled trials, followed by large cohort and cross-sectional studies where higher-level evidence was unavailable. Additional sources were identified by hand-searching reference lists and by consulting professional society and government resources (e.g., CDC, U.S. Department of Veterans Affairs, American Society of Parenteral and Enteral Nutrition). Titles and abstracts were screened by the authors for relevance to older adults (generally ≥65 years), and full texts were reviewed for selected sources; disagreements were resolved by discussion. For the case study, we present an older female who is being evaluated for surgical risks associated with frailty and nutrition before scheduling her surgical procedure.

### 2.1. Case Study

Mrs. Sarah Jones is a 71-year-old female Air Force Veteran referred for right hemicolectomy after diagnosis of nonmetastatic colon adenocarcinoma discovered during evaluation of iron-deficiency anemia. Her medical history includes heart failure with preserved ejection fraction, chronic kidney disease, osteoarthritis, depression, and a fall without injury six months earlier. She is widowed, lives in her own home, lives alone, uses a cane outdoors, and relies on her daughter for grocery shopping and transportation. Over the preceding three months, she unintentionally lost 15 pounds (9% of usual body weight) and reports early satiety, fatigue, and intake of approximately one small meal and snacks daily. Her stated goal is to remain independent in her apartment and return to caring for her garden. What are the next steps for Mrs. Jones?

### 2.2. Screening: Frailty/Physical Performance

Frailty screening should be targeted at older adults with a history of multimorbidity, falls, weight loss, poor nutritional intake, slowed walking, functional decline, polypharmacy, cognitive concerns, recurrent hospitalization, or increased caregiver needs [7]. The Risk Analysis Index (RAI) provides a multidomain frailty-risk estimate based on the deficit accumulation frailty model that reliably projects short-term and long-term mortality in both surgical and nonsurgical adult populations [12,13,14,15,16,17]. The Risk Analysis Index (RAI) is a weighted, 14-item instrument, and scores range from 0 to 81 points (higher scores indicate greater frailty and risk), calculated based on age, sex, cancer status, co-morbid medical conditions, living status, nutrition status, cognitive status, and ability to perform activities of daily living (ADLs). RAI scores of ≥37 indicate the patient is frail, and ≥45 indicate that the patient is very frail [17]. Administration of the RAI takes about 3 min, using self-report or caregiver report supplemented by clinical judgment. Items related to weight loss, appetite, and cognitive decline are often hard for frail patients to answer, so the clinician may need to assess these questions based on their physical examination and health assessment. It was developed and initially validated for prospective preoperative use and is feasible for large-scale clinical screening and more recently calibrated to identify more precise frailty risk [14,17] (see Table 1. Risk Analysis Index (RAI)). Dicpinigaitis et al. (2024) [13] developed and validated an ICD-10 code-based adaptation of the Risk Analysis Index (RAI-ICD), demonstrating that frailty can be reliably identified using administrative health data, thereby enabling large-scale automated screening and risk stratification of surgical and hospitalized patients. They also found that increasing RAI-ICD scores (indicating increased frailty) were reportedly associated with an increase in hospital length of stay, hospital charges, and in-hospital mortality [13].

In addition to RAI, there are other tools that can be used by the clinician to screen for frailty [18,19]. The FRAIL scale is another screening tool that provides a rapid patient-reported screen score based on a mnemonic “FRAIL” (F = fatigue, R = resistance, A = ambulation, I = illnesses, and L = loss (weight loss)) [18,19]. The scoring for this screening test is 0–5 (0 = robust/not frail) and 1–2 = pre-frail, and 3–5 = frail. A positive frailty screen does not independently establish a frailty diagnosis; it should prompt a Comprehensive Geriatric Assessment (CGA) to identify contributors such as sarcopenia, malnutrition, adverse medication effects, acute illness, cognitive impairment, depression, mobility limitation, and social or environmental barriers [20,21,22]. The Clinical Frailty Scale (CFS) is a validated tool that evolved from the Canadian Study of Health and Aging and is frequently used in clinical settings for clinicians to assess frailty and fitness based on clinical judgment and clinician evaluation of a patient. The CFS is scored 1–9 (1 = very fit and 9 = terminally ill), where clinicians can use the tool to summarize the overall level of fitness or frailty of an older adult [22,23].

Several performance tests can also be completed to give additional insight to the clinical team [12,13,18,19,20,24,25,26,27]. Gait speed and the Timed Up and Go test assess mobility and can identify frailty risk; hand-grip strength and chair-rise testing assess muscle strength and physical performance, including possible sarcopenia [24,25,27]. Table 2 highlights selected frailty and performance assessments that may be used to evaluate frailty and functional performance in older adults.

For our case study, Mrs. Jones’ frailty screening is completed by her clinician at pre-surgical consultation using the Risk Analysis Index (RAI) [17]. The clinician determines that the RAI screen is elevated (61), which indicates she is at risk for frailty [17]. The frailty/prehabilitation evaluation identifies slowed gait speed, difficulty completing five chair rises without using her arms, reduced grip strength, a positive cognitive screen requiring further evaluation, and nutrition risk. As she is assessed further, it is determined that she has 5/5 phenotypic criteria (Fried Criteria) for the diagnosis for frailty syndrome, including unintentional weight loss (about 10 pounds or 5% in one year), self-reported exhaustion, weakness (low grip strength, measured with a dynamometer), slow walking speed, and low physical activity [2]. These findings prompt her surgical team to refer her for a prehabilitation evaluation and intervention before surgical intervention. See Table 3 for the RAI calculations for this fictional case study example.

### 2.3. Screening: Malnutrition Risk

Routine malnutrition risk screening is recommended for all older adults, including those who are overweight or obese, because body size alone does not reliably reflect muscle mass, nutrient adequacy, or nutritional reserves [30,31]. Malnutrition risk screening in primary care and pre-operative settings should be routine for older adults, especially those with chronic disease, recent hospitalization, functional decline, frailty, unintentional weight loss, food insecurity, dysphagia, or polypharmacy [30,31]. The frequency of malnutrition screening is based on risk factors (unintentional weight loss, reduced appetite, dental/oral problems, after hospitalization/emergency visits, or other change in health status) that are present, but it is generally recommended to be performed on a routine, repeated basis in primary care and during chronic disease management [30,31]. The Registered Dietitian is particularly important in completing a detailed dietary assessment and nutrition-focused physical examination [30,31]. However, Registered Nurses and other frontline clinicians are often the first to screen patients for nutrition risk and detect red flags, including declining meal intake, unexplained weight change, increased fatigue, functional loss, repeated falls, denture problems, or inability to prepare meals [7]. Screening identifies patients needing further assessment; it is not itself a diagnosis of malnutrition. A positive result should trigger a comprehensive nutrition assessment, ideally by a registered dietitian, followed by an individualized nutrition care plan [32,33].

Several validated tools can identify malnutrition risk in primary care and community settings [33,34,35]. The Mini Nutritional Assessment–Short Form (MNA-SF) is particularly suited for adults aged 65 years and older because it evaluates appetite or intake decline, weight loss, mobility, recent acute illness or stress, cognition or depression, and BMI or calf circumference; scores of 12–14 indicate normal nutritional status, 8–11 indicate risk of malnutrition, and 0–7 indicate malnutrition [33,35]. The MNA-SF can be completed in less than five minutes. It also allows calf circumference to replace BMI when accurate height or weight cannot be obtained [33,35]. The Malnutrition Universal Screening Tool (MUST) is another method for screening for malnutrition that incorporates BMI, unintentional weight loss, and the acute-disease effect (defined as little to no nutritional intake for more than 5 days). Scores are combined to classify patients as low risk (0), medium risk (1), or high risk (≥2) for malnutrition, guiding the need for nutritional intervention and follow-up [33]. The Malnutrition Screening Tool (MST) is a rapid option that focuses on recent unintentional weight loss and poor appetite or reduced intake; a score of 2 or greater indicates nutrition risk and should prompt comprehensive assessment and intervention [33,36]. In a 2025 prospective study of hospitalized older adults, the Mini Nutritional Assessment–Short Form (MNA-SF) demonstrated the highest overall accuracy among the MNA-SF, Malnutrition Screening Tool (MST), and Malnutrition Universal Screening Tool (MUST), when evaluated against GLIM-based malnutrition criteria [34].

In our case study, a Registered Nurse assessed Mrs. Jones’ malnutrition risk using the Malnutrition Screening Tool (MST). Her MST score indicated that she was at risk for malnutrition (MST = 3, due to weight loss and poor appetite) [33,36]. An MST score of greater than 2 prompted referral and nutrition assessment by the Registered Dietitian.

### 2.4. Nutrition Assessment/Diagnosis

A clinical nutrition assessment should include: (1) recent and long-term weight history, including percentage and time course of unintended weight loss; (2) usual dietary pattern, food access, meal frequency, appetite, and estimated protein and energy intake; (3) symptoms affecting intake, such as nausea, early satiety, constipation, diarrhea, pain, xerostomia, chewing difficulty, dysphagia, or altered taste; (4) functional ability to obtain, prepare, and consume food; (5) mood, cognition, social support, alcohol use, financial resources, and housing stability; (6) comprehensive medication reconciliation including medications, dietary supplements, review for medications that affect appetite, gastrointestinal tolerance, taste, alertness, or nutrient absorption; and (7) nutrition-focused physical examination to evaluate subcutaneous fat loss, muscle wasting, fluid accumulation, and functional status. Objective measures, when available, such as handgrip strength, gait speed, chair-rise performance, bioelectrical impedance analysis, or other assessments of muscle mass [30,32,37,38].

In the United States, the Academy of Nutrition and Dietetics (AND) and American Society for Parenteral and Enteral Nutrition (ASPEN) recommend diagnosing adult malnutrition when at least two of six clinical characteristics are present [37]. These 6 characteristics include insufficient energy intake, unintentional weight loss, loss of muscle mass, loss of subcutaneous fat, fluid accumulation (edema), and reduced functional status (often assessed by hand-grip strength). The diagnosis should also identify the underlying context: starvation-related malnutrition with minimal inflammation; chronic disease-related malnutrition, such as that occurring with cancer, pulmonary disease, kidney disease, heart failure, or inflammatory bowel disease; or acute disease/injury-related malnutrition associated with conditions such as sepsis, trauma, major surgery, or burns [37].

For our fictional case study patient, Mrs. Jones, the ASPEN/AND Malnutrition Diagnosis Criteria were used to determine the presence of severe chronic disease- or condition-related malnutrition. Mrs. Jones has lost 9% of her weight (criteria of >7.5% weight loss in three months indicative of severe malnutrition). Her three-day diet recall indicated an energy intake of <75% of estimated needs for greater than one month (an indicator for either moderate or severe malnutrition). Her Nutrition Focused Physical Exam (NFPE) revealed severe muscle depletion at her clavicles, scapula, shoulders, and temples. Severe fat loss was noted at triceps, ribs, and orbital fat pads. A diagnosis of malnutrition can be made if two or more indicators are found using the ASPEN/AND Nutrition Diagnosis Guide. Mrs. Jones had four indicators that meet criteria to diagnose severe malnutrition: severe muscle loss, severe fat loss, >7.5% weight loss in three months, and <75% energy intake greater than one month.

Alternatively, some clinicians utilize the Global Leadership Initiative on Malnutrition (GLIM) to establish the diagnosis and severity of malnutrition [32,38]. The GLIM framework recommends a two-step process for diagnosing malnutrition: first, identify risk using a validated screening tool; second, perform a nutrition assessment [32,38]. A GLIM diagnosis requires at least one phenotypic criterion and one etiologic criterion. Phenotypic criteria include involuntary weight loss, low body mass index, and reduced muscle mass. Etiologic criteria include reduced food intake or impaired nutrient assimilation and disease burden or inflammation [32,38]. Malnutrition severity is graded using phenotypic criteria such as Stage 1 (moderate) or Stage 2 (severe) [32,38]. This approach emphasizes that malnutrition is not diagnosed solely by weight or body mass index. This is important because the patient with obesity may experience substantial involuntary weight loss, muscle depletion, inadequate intake, and inflammation and still meet criteria for malnutrition. Using the GLIM framework, our fictional case study patient met three phenotypic criteria including 9% unintentional weight loss in three months, BMI of 19.4 (current weight 54.4 kg and height 5 feet, 6 inches), and muscle depletion (as described above). In addition, two etiologic criteria were met, including reduced intake for about four months (three-day recall averaging ~680 kcal and 24 g protein daily, roughly 35 to 40% of estimated needs), and chronic disease burden with inflammation from malignancy, chronic kidney disease, and heart failure. For a patient aged 71 years, the reported BMI of 19.4 kg/m^2^ meets the standard GLIM BMI criterion for Stage 2 (severe) malnutrition. This raises her risk of postoperative complications and functional decline, which supports early nutrition intervention to help her remain independent at home [38].

### 2.5. Comprehensive Geriatric Assessment

Comprehensive Geriatric Assessment (CGA) is a structured, interdisciplinary process that can strengthen both identification and management of frailty [20,21,39,40,41]. Rather than relying on frailty screening alone, CGA evaluates the interrelated contributors to vulnerability, including multimorbidity, medication burden, functional status, mobility and falls, cognition and delirium risk, mood, nutrition and sarcopenia, oral health, continence, social support, caregiver capacity, home safety, and patient goals. CGA is particularly appropriate in older adults with frailty, multimorbidity, functional decline, recurrent falls, cognitive concerns, polypharmacy, or other complex geriatric needs [41]. A 2024 systematic review and meta-analysis of 18 randomized controlled trials found that CGA was associated with lower mortality and a reduced risk of decline in activities of daily living among hospitalized frail older adults [39]. Findings should be translated into an individualized, coordinated care plan that includes medication optimization, physical and occupational therapy, resistance and balance exercise, nutrition support, oral-health or swallowing referral, fall-prevention strategies, caregiver education, and social-service linkage [3,7,21,39,41,42]. Table 4 highlights the key aspects and assessments that are completed during the CGA process.

Poor oral health and declining oral function are associated with a higher risk of physical frailty in older adults [42,43]. Problems such as tooth loss, dental pain, poorly fitting dentures, dry mouth, weak chewing, and swallowing difficulty can reduce food intake and dietary variety, leading to malnutrition, sarcopenia, weakness, and loss of independence. A systematic review of 39 articles found that poor oral health (oral health status deterioration and few remaining teeth) was associated with frailty [42,43]. For frail or nutrition-risk older adults, it is suggested to include oral-health questions in the comprehensive geriatric assessment, including questions about tooth or mouth pain, dry mouth, denture fit, changes in food texture or avoidance, chewing or swallowing difficulty, and ability to perform daily mouth care. Addressing denture problems, medication-related dry mouth, dental disease, dysphagia, and nutrition issues may support adequate intake, function, and prevention or mitigate frailty [31].

Medication review is an important part of comprehensive assessment for all adults, but particularly for older adults due to the potential for polypharmacy [44,45,46]. When taking multiple medications at the same time throughout the day, medication-related adverse effects can reduce intake, impair nutrient absorption, alter oral function, or worsen function and independence in older adults. These can all lead to problems with malnutrition and frailty. In a frail older adult, reduced physiologic reserve and altered pharmacokinetics increase susceptibility to medication-related harm. This potential risk is compounded during and after surgery [44,45,46]. Medication review should be a routine part of Comprehensive Geriatric Assessment, particularly after a fall, delirium episode, weight loss, hospitalization, new difficulty with ADLs, or recurrent ED visits [41,44,45,46,47]. Table 5 highlights medication classes that may impair nutrition status or influence frailty in older adults.

For our case study, Mrs. Jones’ medications were reviewed, and it was found that she had duplicate medications (she was taking over-the-counter diphenhydramine for a skin rash and was taking combined Acetaminophen and Diphenhydramine at night to help her sleep). These medications were discontinued, and other non-pharmacologic interventions (guidance on sleep hygiene techniques and education on omitting triggers for dermatitis symptoms) were provided to Mrs. Jones for these issues. Mrs. Jones’ oral health was assessed as part of the comprehensive assessment, and she had no loose teeth or active cavities. She was referred to her primary dentist and dental hygienists for routine follow-up and hygiene care.

### 2.6. Nutritional Interventions

For older adults preparing for surgery, nutrition is a key component of prehabilitation because it can help improve physiologic reserve, support muscle health, and reduce the risk of frailty before the stress of surgery [48,49]. Protein intake is a major modifiable nutritional factor in frailty prevention and management [50]. Protein supports skeletal-muscle protein synthesis, immune function, wound healing, and recovery from illness. Inadequate protein intake can contribute to loss of lean body mass and sarcopenia, weakness, impaired mobility, and frailty [30,48].

Daily protein targets should be matched to clinical status, renal function, and treatment goals [30,48]. Several studies and practice guidelines recommend that older adults consume at least 1.0–1.2 g/kg/day of protein, with individual adjustment based on nutritional status, physical activity, disease burden, and tolerance [30,48]. For individuals with frailty, malnutrition, acute illness, rehabilitation needs, or other catabolic stressors, higher intake may be considered when clinically indicated. Individualized protein targets should be individualized based on health status, nutrition status, physical activity, disease burden, and/or renal/hepatic disease [30,48]. Evidence suggests that protein is most effective when incorporated into a multicomponent plan (often with exercise or resistance training) rather than provided in isolation [3]. In a 12-week randomized controlled trial of undernourished prefrail and frail older adults, protein supplementation at 1.5 g/kg/day improved skeletal muscle mass and gait speed compared with 0.8 g/kg/day; the benefit was less clear at 1.2 g/kg/day [50]. A systematic review and meta-analysis similarly found that protein interventions can augment the benefits of resistance exercise on appendicular lean mass and handgrip strength. Importantly, in pooled comparisons without resistance exercise, protein alone did not significantly improve total lean body mass, appendicular lean mass, grip strength, or knee-extension strength, highlighting the importance of multi-component interventions [51].

A prospective cohort study of older Chinese adults found that greater dietary protein-source diversity, including both animal and plant sources, was associated with lower frailty risk [52]. Protein prescriptions for older adults should generally be based on actual body weight, although they may need to be tailored based on clinical judgment and dietitian input, especially for those with obesity, edema or ascites, amputations, impaired kidney or liver function, or substantial recent weight changes [53]. Protein should be distributed across meals through the day, with approximately 25–30 g of high-quality protein per meal adjusted for body size, appetite, renal function, and the overall daily target [48]. Appropriate sources may include fish, poultry, eggs, dairy foods such as Greek yogurt, milk, and cottage cheese, lean meats, soy foods, legumes, protein-fortified foods, and oral nutrition supplements when usual intake is insufficient. For frailty prevention, protein intake should be combined with progressive resistance, balance, and functional exercise, since protein supplementation alone has less consistent benefit for muscle strength and physical function [30,53].

A growing body of evidence suggests that adherence to a Mediterranean-style dietary pattern is associated with lower rates of frailty and may help optimize surgical readiness [54,55,56]. This dietary pattern emphasizes vegetables, fruits, legumes, whole grains, nuts, seeds, olive oil, and fish, while limiting highly processed foods, added sugars, and processed meats. These foods provide fiber, healthy fats, antioxidants, and other bioactive compounds that may reduce inflammation and oxidative stress, which are important contributors to frailty and poor postoperative recovery [56,57,58].

The Mediterranean dietary pattern may also promote a healthier gut microbiome, which has been increasingly linked to physical resilience and frailty in older adults [57,58,59]. Higher fiber intake supports beneficial gut bacteria that produce short-chain fatty acids, compounds involved in maintaining intestinal health and regulating inflammation. Emerging evidence suggests that Mediterranean-style eating may improve gut microbial diversity and is associated with lower frailty and better overall health outcomes [60,61,62]. As part of a comprehensive prehabilitation program, Mediterranean-style nutrition should be individualized to accommodate cultural preferences, food access, budget, oral health, swallowing ability, kidney function, and other medical considerations to ensure it is both practical and sustainable before surgery [56].

Several components of the Mediterranean diet may be relevant for pre-surgical older adults. For example, olive oil and omega-3 fatty acids from seafood such as salmon, sardines, and trout may support muscle function and help modulate inflammatory processes [59]. Polyphenol-rich foods, including berries, vegetables, legumes, nuts, tea, and olive oil, may further support healthy aging by reducing oxidative stress and preserving physical function [57,59]. While this evidence is limited, there is a growing body of evidence supporting a nutrient-dense, plant-forward eating pattern combined with adequate protein intake as an important strategy for optimizing functional status [49,50,51,54,55,56,62].

Clinicians should first address food intake, energy adequacy, meal access, nutrition-impact symptoms, and individualized protein distribution [30,31]. The International Conference on Frailty and Sarcopenia Research guidelines conditionally recommend protein and/or calorie supplementation for people with frailty who have weight loss, cannot attain required calories, macronutrients, or micronutrients, or have documented undernutrition [1]. Oral nutrition and/or protein supplements may be appropriate when food-first strategies are insufficient or clinically significant weight loss or undernutrition is present [30,31]. For an older adult with low appetite, strategies may include adding Greek yogurt, peanut butter, cheese, powdered milk, or oral nutrition supplements to foods already accepted by the patient.

For frailty prevention, protein intake should be combined with progressive resistance, balance, and functional exercise to preserve or improve strength, muscle mass, mobility, and physical performance [29,30,53]. Resistance training is the principal exercise modality for improving muscle mass and strength; combining resistance and aerobic exercise can provide additional physical-function benefits, especially during prehabilitation before surgery [29,63,64].

### 2.7. Interdisciplinary Approach

Frailty management requires a multi-pronged, interdisciplinary approach [29,49]. Resistance exercise, physical therapy, balance training, mobility work, and occupational therapy in conjunction are essential because food intake and nutritional therapy without maximizing movement and mobility will not adequately reverse functional decline [29,49]. A 2026 meta-analysis found that multicomponent exercise and nutrition interventions can improve frailty status in older adults, with combined approaches generally outperforming nutrition-only strategies [29]. The FRAILMERIT multicenter clinical trial likewise reported that a primary-care-led intervention using an interdisciplinary team and combining exercise, nutrition recommendations, and frailty education improved frailty status or physical function among prefrail or frail community-dwelling older adults [64]. These findings reinforce that nutrition builds capacity, but progressive activity helps translate nutritional support into improved strength, mobility, and independence.

A coordinated interdisciplinary frailty care plan may include: (1) dietitian referral for malnutrition assessment and individualized protein-energy targets; (2) physical therapy for gait, balance, lower-extremity strength, fall-risk reduction, and individualized resistance training; (3) occupational therapy for meal preparation, adaptive equipment, energy conservation, and activities of daily living; (4) speech–language pathology for swallowing evaluation and texture-modified recommendations when dysphagia is suspected; (5) dental assessment for pain, ill-fitting dentures, missing teeth, or chewing difficulty; (6) nursing follow-up for weight trends, intake, symptom monitoring, education reinforcement, medication management, and coordination of referrals; or (7) social work involvement for food insecurity, caregiver support, housing instability, transportation, benefits, and community resources [64,65,66].

Additionally, clinicians can use the Age-Friendly Health System (AFHS) 4Ms framework “What Matters, Medication, Mentation, and Mobility” to structure interdisciplinary care [67]. Clinicians asking, “What matters to you?” may uncover priorities such as remaining at home, attending a family event, avoiding hospitalization, being able to cook independently, or maintaining strength for caregiving responsibilities. These priorities can guide realistic nutrition and rehabilitation goals for the patient, such as Mrs. Jones [67]. Potential interdisciplinary and resource support includes outpatient and inpatient dietitian consultation, primary care and geriatric services, caregiver-support services, social work, assistance, dental services, home-based primary care when eligible, rehabilitation services, and specialized frailty or surgical prehabilitation programs. Local food banks, congregate meal sites, meal delivery services, and community-based programs can be helpful to reduce barriers and optimize nutrition and functional interventions [65,66].

### 2.8. Comprehensive Prehabilitation

As we see from Mrs. Jones’ case study, frailty is a significant biologic state for any older person; however, it is particularly consequential in the perioperative setting because surgery may uncover limitations in an older individual’s physiologic reserve and precipitate postoperative complications, prolonged hospitalization, delirium, functional decline, institutionalization, and death [4,10]. Over the past several years, more attention has been paid to surgical risk for older adults, and care pathways such as the Enhanced Recovery after Surgery (ERAS) strategy have been adopted at many health systems as a standard of care for perioperative management of routine surgical patients as well as populations of people who are older, frail, and malnourished [49,63]. ERAS pathways provide an important structure for operationalizing many prehabilitation goals. For example, nutritional optimization before surgery supports ERAS objectives related to metabolic preparation and early postoperative feeding, while exercise training and functional conditioning complement ERAS recommendations for early mobilization. Similarly, medication review, delirium-risk assessment, smoking cessation, and patient education are foundational components of both comprehensive prehabilitation and modern ERAS programs [16,49,63]. Within this context, identifying patients who are at significant frailty risk and tailoring interventions to meet their needs is essential [49,63]. Accordingly, frailty assessment should occur early enough in the surgical decision process to inform the choice of treatment, clarify the patient’s goals, and permit meaningful optimization rather than serving only as a late-stage prediction tool [14,17].

As an example of a programmatic approach to identifying persons at risk for post-operative complications related to frailty, the U.S. Department of Veterans Affairs (VA) created a program called “Surgical Pause” that is a perioperative quality-improvement model developed to identify frailty before elective surgery and use that information to improve shared decision-making and prehabilitation [16,68]. The VA disseminated the Surgical Pause through its Diffusion of Excellence framework as a standardized approach to identify high-risk patients and connect them with targeted multidisciplinary care [16,68]. The empirical foundation for the model includes the original Frailty Screening Initiative [14,16,68]. In a prospective cohort, implementation of routine preoperative RAI screening was associated with a decrease in overall 30-day mortality from 1.6% to 0.7%. Among patients identified as frail, 30-day mortality decreased from 12.2% to 3.8% following implementation [15]. Varley and colleagues (2023) conducted a quality-improvement study of 50,463 patients undergoing major operations and found that routine RAI assessment linked to an electronic health-record alert was associated with an 18% reduction in the adjusted odds of one-year postoperative mortality; one-year mortality declined from 3.9% to 3.3% overall and from 20.2% to 16.0% among frail patients [69]. Embedding frailty screening into workflow and acting on results (goals-of-care discussions, optimization, care pathways) is associated with substantial reductions in short- and mid-term mortality, especially for frail patients [13,15,69]. The strength of this evidence is weak in that these findings are based on observational studies and quality improvement initiatives; therefore, more research is needed using stronger methodologies and within multiple settings, health systems, and populations.

Within the VA Surgical Pause model, an elevated RAI score triggers a deliberate “pause” before proceeding [16]. The pause is not synonymous with cancellation or denial of surgery. Rather, it creates a structured opportunity to consider whether surgery is likely to help the individual achieve what matters most, whether nonoperative management is preferable, and whether a time-limited course of prehabilitation could improve readiness for surgery. The model emphasizes that a patient’s risk estimate should be paired with shared decision-making, advance care planning, and practical steps to reduce modifiable risk [16,68].

The VA Surgical Pause model uses interdisciplinary review to translate screening findings into an actionable plan. Depending on patient needs, the team may include surgery, anesthesiology, geriatrics, nursing, nutrition, physical therapy, occupational therapy, speech–language pathology, pharmacy, social work, palliative care, and behavioral health. This approach recognizes that frailty is not a single deficit and that optimization must address physical, nutritional, cognitive, psychosocial, and medical aspects of person-centered care [16,68].

A comprehensive prehabilitation such as the surgical pause program for an older adult may include: (1) functional assessment, including activities of daily living and instrumental activities of daily living; (2) cognitive screening and evaluation of delirium risk; (3) malnutrition screening, nutrition assessment, and individualized protein-energy planning; (4) physical performance testing, such as gait speed, timed up-and-go, chair-rise testing, step testing, and grip strength; (5) assessment of durable medical equipment needs to promote home and community safety; (6) medication reconciliation and optimization, including assessment of medications that increase delirium, fall risk, bleeding, orthostasis, or poor intake risk; (7) pulmonary, cardiovascular, and anesthesia assessment, as indicated; (8) palliative-care consultation to clarify goals of care, treatment preferences, and acceptable trade-offs; or (9) social work assessment of caregiver availability, transportation, food access, housing stability, and discharge needs [16,68,69,70,71].

Evidence for prehabilitation in older and frail surgical populations is promising but heterogeneous; studies support its feasibility and suggest potential reduction in postoperative complications and improvement in functional outcomes, while the ideal components, intensity, duration, and target population are continuing to be defined [49,63,69,70]. The appropriate duration of prehabilitation depends on surgical urgency, diagnosis, baseline status, and response to intervention [70]. Elective surgical procedures may allow several weeks for frailty and functional improvement, whereas urgent or oncologic procedures may require an abbreviated but intensive plan. Importantly, prehabilitation prior to surgery can lead to more than one appropriate pathway: proceeding to surgery after optimization, selecting an alternative intervention, continuing conservative management, or deciding that surgery is inconsistent with the patient’s goals and acceptable outcomes [70,71].

### 2.9. Management Plan

As Mrs. Jones visits the prehabilitation clinic, the interdisciplinary team develops a four-week, time-limited optimization plan while the oncology and surgical teams confirm that a brief delay is clinically acceptable. Based on her current body weight of 54.4 kg, her estimated energy requirements were approximately 1630 kcal/day using the ESPEN recommendation of 30 kcal/kg/day for older adults [30]. Given her colorectal malignancy, frailty, inflammatory burden, and evidence of malnutrition, protein requirements were estimated at 1.2–1.5 g/kg/day, equivalent to approximately 65–82 g protein daily [30,71]. Compared with her observed intake of approximately 680 kcal and 24 g protein per day, Mrs. Jones was meeting only 38–43% of estimated energy needs and 30–37% of protein needs, supporting the diagnosis of moderate disease-related malnutrition with inflammation. The management plan includes the following:

- Dietitian-directed energy and protein goals, targeting 30 kcal/kg/day and 1.2–1.5 g/kg/day of protein if compatible with renal and overall clinical status [30,71].
- Small, frequent meals and high-protein foods tailored to her tolerance and preferences, including Greek yogurt, eggs, fortified oatmeal, nut butter, beans, fish, poultry, and high-protein oral nutrition supplements between meals.
- Encourage high-protein and nutrient-rich snacks between meals to enhance calories, protein, and nutrients.
- Manage early satiety and constipation with small, frequent meals (including fiber and protein sources) and adequate hydration as tolerated.
- Review medications (especially those that may suppress appetite or contribute to dizziness).
- Physical therapy twice weekly plus a home program focused on sit-to-stand practice, progressive lower-extremity strengthening, walking, balance, and breathing exercises.
- Anesthesia pre-surgical evaluation and medication plan addressing perioperative volume status and delirium-risk reduction strategies.
- ERAS education, including counseling regarding expected recovery milestones, pain management, nutrition goals, early mobilization, and discharge planning to improve patient engagement and preparedness [63].
- Carbohydrate loading and perioperative fasting management as appropriate, following institutional ERAS protocols and considering blood glucose management needs [63].
- Optimization of anemia, hydration, and glycemic control before surgery to reduce postoperative complications and support recovery [63].
- Multimodal opioid-sparing pain management plans to minimize postoperative opioid exposure, reduce delirium risk, and facilitate early mobility [63].
- Preoperative pulmonary optimization, including deep breathing exercises and incentive spirometry instruction to reduce postoperative pulmonary complications [63].
- Social work coordination of meal delivery, transportation, daughter’s participation in discharge planning, and identification of post-discharge support.
- Encourage dental hygiene and maximizing dental care to enhance oral function, promote chewing of foods, and improve oral health.
- Palliative-care-supported shared decision-making to review prognosis, anticipated recovery, acceptable outcomes, and the possibility of nonoperative management should her health decline or goals change.

Nutrition and functional interventions may fail if practical barriers are not addressed. Common obstacles include food or housing insecurity, limited income, transportation difficulties, cognitive impairment, depression, social isolation, reduced mobility, chronic pain, inability to prepare meals, poor dentition, dysphagia, low health literacy, language barriers, and lack of caregiver support.

For Mrs. Jones, the team communicates with her daughter (primary family member, healthcare surrogate, and caregiver) and discusses any barriers or concerns that they have. Both Mrs. Jones and her daughter were concerned about transportation to/from medical appointments, as Mrs. Jones does not currently drive and it is difficult for her daughter to take her to all appointments. A plan was developed to connect Mrs. Jones with ride-share services in her community using a commercial ride-share application that her daughter assists with. This plan was agreeable to both Mrs. Jones and her daughter.

### 2.10. Monitoring/Evaluation

After four weeks, Mrs. Jones stabilized her weight (gained 5 pounds), increased average protein and calorie intake, improved chair-rise performance and walking tolerance, and showed greater confidence with the home exercise plan. Her daughter confirms improved meal consistency and agrees to provide additional support after discharge following surgery. The multidisciplinary team reviews the remaining risks and determines that surgery remains consistent with Mrs. Jones’ goals. She proceeds with an enhanced perioperative plan that includes delirium-prevention measures, early mobilization, nutrition follow-up, and discharge planning. Mrs. Jones continues a post-operative medical oncology plan, rehabilitation, nutrition, and exercise plan coordinated with her interdisciplinary healthcare team. Mrs. Jones proceeds with surgery under an Enhanced Recovery After Surgery (ERAS) pathway where she will have shortened preoperative fasting (clear liquids permitted up to 2 h before surgery and solids up to 6 h before surgery), multimodal analgesia with opioid-sparing strategies, goal-directed fluid management, early oral nutrition, early mobilization beginning on the day of or first day after surgery, delirium-prevention measures, and coordinated discharge planning. The integration of frailty-informed prehabilitation with ERAS principles is intended to reduce postoperative complications, preserve functional independence, shorten recovery time, and support a return to baseline quality of life. This fictional case demonstrates in an illustrative way that the purpose of preoperative frailty screening is not to label a patient as “too frail” for care. It is to identify risk early, align treatment with the person’s goals, address modifiable vulnerabilities, and prepare the patient and caregivers for the likely recovery trajectory. Follow-up care on an ongoing basis with her interdisciplinary geriatric team will be important to ensure comprehensive, holistic care throughout her life.

## 3. Limitations

This review has limitations, as although the case study depicts a common patient scenario, it is fictional and illustrative. As a narrative review, the authors did not comprehensively evaluate all the literature or perform a formal risk-of-bias assessment, so relevant studies may have been missed, and the review reflects the authors’ judgment. Additionally, much of the evidence on dietary patterns and individual nutrients is observational and cannot establish causality. More research is needed to determine the best approaches for maximizing outcomes.

## 4. Conclusions

Frailty and malnutrition are closely interconnected yet potentially reversible conditions that contribute to declines in muscle mass, physical function, independence, and overall resilience among older adults. Early changes in appetite, dietary intake, weight, oral health, strength, mobility, cognition, mood, and social support may indicate increasing vulnerability. Therefore, routine screening for both malnutrition and frailty should be incorporated into the care of older adults at risk. Positive screening results should lead to a comprehensive geriatric assessment and the development of an individualized care plan that addresses the medical, nutritional, functional, medication-related, and social factors contributing to risk, rather than relying solely on body weight or a single screening tool.

Optimal management requires an interdisciplinary approach involving dietitians, nurses, physicians, pharmacists, physical and occupational therapists, speech–language pathologists, dentists, social workers, and palliative care specialists. Equally important are supports that address practical barriers such as food insecurity, transportation needs, caregiving assistance, and meal preparation. Personalized nutrition interventions focused on adequate energy and protein intake, food-first approaches, and adoption of a Mediterranean-style dietary pattern should be combined with progressive resistance and functional exercise, medication optimization, oral health and dysphagia management, and social support services.

Mrs. Jones’ fictional case demonstrates the significant impact that frailty and malnutrition can have on surgical risk in older adults. Early screening identified several modifiable concerns, including unintentional weight loss, inadequate nutritional intake, reduced physical performance, and functional limitations. Through a comprehensive prehabilitation program incorporating nutritional optimization, exercise, medication review, social support, and shared decision-making, her readiness for surgery improved while supporting her goal of preserving independence and quality of life. In this case, frailty screening informed a multidisciplinary prehabilitation plan that was subsequently integrated into an ERAS pathway, creating a comprehensive perioperative strategy focused on optimizing physiologic reserve, reducing surgical stress, and promoting recovery. Ultimately, integrating individualized nutrition care, exercise, interdisciplinary collaboration, and patient-centered care strategies offers the potential to maintain strength, function, independence, and quality of life in older adults. Continued research is needed to further strengthen the evidence base and optimize interventions for this vulnerable population.

## Figures and Tables

**Table 1:** Risk Analysis Index (RAI).

RAI Domain	#	RAI Item	RAI Response Options
**Demographics**	1	Age	Years (numeric)
	2	Sex	Male/Female
**Social history**	3	Lives in a nursing home, skilled nursing facility, or assisted living?	Yes/No
	3.1	*(if yes)* Where does the patient reside?	Nursing home/Skilled nursing facility/Assisted living
	3.2	*(if yes)* When did they begin living there?	<3 months ago/3 months to 1 year ago/>1 year ago
**Medical history**	4	Ever seen a nephrologist or history of kidney problems?	Yes/No
	4.1	*(if yes)* Kidney stones or another problem?	Kidney stones/Other/Both
	5	Chronic congestive heart failure?	Yes/No
	6	Shortness of breath at rest or with minimal activity?	Yes/No
	7	Diagnosed or treated for cancer in the past 5 years?	Yes/No
	8	Unintentional loss of 10+ pounds in the past 3 months?	Yes/No
	9	Poor appetite currently?	Yes/No
	10	Harder to remember things or organize thoughts in the past 3 months?	Yes/No
**Activities of daily living**	11	Mobility	Gets around without help/Needs cane, walker, or scooter/Needs help from others around house or neighborhood/Needs help getting in or out of a chair/Totally dependent
	12	Eating	Plans and prepares own meals/Needs help planning/Needs help preparing/Needs help eating/Totally dependent
	13	Toileting	Uses toilet without help/Needs help getting to or from toilet/Needs help with toilet paper/Needs bedpan or urinal with help/Totally dependent
	14	Personal hygiene	Bathes without prompting or help/Bathes without help when prompted/Needs help preparing tub or shower/Needs help with some washing/Totally dependent

Note. Adapted from Hall et al. (2017) [14] and Arya et al. (2020) [17].

**Table 2:** Frailty and Performance Assessments.

Test	What it Assesses	Typical Normal Result	Concern for Frailty	Important Notes
**Gait Speed**	Performance measure. Usual walking speed; mobility and physiologic reserve	≥0.8 m/s	<0.8 m/s	Use usual assistive device if normally used. Slow gait is a strong marker of vulnerability [26].
**Timed Up and Go (TUG) Screening Test**	Screening test for risk for falls and impaired mobility, transfers, gait, turning, balance	≤12 s	>12 s	Patient rises from chair, walks 3 m, turns, returns, and sits. It is a frailty/falls screen, not a stand-alone diagnostic test. [27].
**Five-times chair-rise test (Five-Times Sit-to-Stand Test) (5 × STS)**	Performance measure. Lower-extremity strength	≤15 s	>15 s, inability to complete, or need to use arms	Use a standard-height chair; avoid if medically unsafe. A prolonged time suggests low lower-extremity strength. May be difficult to complete 5 times in a frail older person [25].
**Hand-grip strength**	Performance measure. Overall muscle strength	≥27 kg men; ≥16 kg women	<27 kg men; <16 kg women	Dynamometer type and protocol should be standardized, usually measured three times in the dominant hand [24].
**Short Physical Performance Battery (SPPB)**	Balance, 4-m gait speed, and chair stands	10–12/12	≤8/12 commonly indicates poor performance; 0–3 (severe limitation)	Combines balance, 4-m gait speed, and chair stands. Useful for tracking change over time and rehabilitation response [28,29].
**Clinical Frailty Scale**	Frailty classification (clinical judgment). Global clinical judgment of fitness, comorbidity, cognition, and function	Score 1–4 (4 = vulnerable) (9-point scale)	Score ≥5 on the 9-point scale generally indicates frailty	If positive for frailty, complete comprehensive geriatric assessment (CGA), discuss goals of care, and create an individualized multidisciplinary plan [22,23].
**Risk Analysis Index (RAI)**	Frailty risk screening (surgical settings) Multidomain frailty risk: function, cognition, nutrition-related symptoms, comorbidity, and residence	Score < 37	≥37 = frail ≥45 = very frail	If positive for frailty, complete CGA, medication/nutrition review, and rehabilitation or prehabilitation planning [14,17].
**FRAIL Scale**	Frailty screening (self-report) tool; evaluates FRAIL (Fatigue, Resistance, Ambulation, Illnesses, and Loss of weight).	0 (robust)	1–2 = prefrail; ≥3 = frail	One point each for fatigue, resistance, ambulation, illnesses, and weight loss. If positive for frailty, evaluate potentially reversible contributors and begin targeted intervention [19].

**Abbreviations:** CGA = Comprehensive Geriatric Assessment; ICD = International Classification of Diseases; PT = physical therapy; OT = occupational therapy; RAI = Risk Analysis Index.

**Table 3:** Fictional Case Study Example: RAI Calculator—United States Department of Veterans Affairs.

Fictional Case Study Example RAI Calculator—United States Department of Veterans Affairs	
Variable	Score
Sex:	0
Cancer Status:	Yes
Age*Cancer Status:	34
Weight Loss:	4
Poor Appetite:	4
Renal Insufficiency:	8
Chronic/Congestive Heart Failure:	5
Shortness of Breath:	0
Dependent Living:	0
Cognitive Decline:	Yes
ADL**Cognitive Decline:	6
Mobility:	Needs help from a cane, walker, or scooter
Eating:	Can plan and prepare his/her own meals
Toileting:	Can use the toilet without help
Personal Hygiene:	Can shower or bathe without prompting or help
RAI Score:	61
RAI Score (without Cancer):	49

*: Age/Cancer interact to adjust the final RAI score: Cancer history increases the RAI score;**: ADL/Cognitive Decline Status combined is scored from 0–16 based on 4 functional domains (mobility, eating, toileting, hygiene). If cognitive decline is present, independent ADL scores mathematically adjust the RAI risk score.

**Table 4:** Comprehensive Geriatric Assessment.

Domain	Key Questions or Assessments
Medical	Ask about multimorbidity, assess frailty, pain, sensory impairment, continence, wounds, falls, potentially inappropriate medications, and medication burden
Functional	Activities of daily living (ADLs), instrumental activities of daily living (IADLs), gait, transfers, balance, mobility aids, and rehabilitation potential
Cognition and mood	Delirium risk, cognitive impairment, depression, anxiety, sleep, and decision-making capacity
Nutrition	Weight history, intake, appetite, dysphagia, dentition, food access, malnutrition, and sarcopenia risk
Medication review	Indications, adverse effects, interactions, anticholinergic/sedative burden, adherence, and deprescribing opportunities
Social and environmental	Caregiver capacity/strain, finances, transportation, isolation, home hazards, and access to community resources
Goals of care	Patient priorities, prognosis, advanced directives, 4M’s, surrogate decision makers, and preferred sites of care (clinic, home, hospital, rehabilitation, long-term care, etc.)

Note: Adapted from Kumlehn et al. (2025) [41].

**Table 5:** Medications that Can Impair Nutrition and/or Impact Frailty in Older Adults.

Medication/Class	Potential Nutrition-Related Effects	Potential Frailty-Related Considerations	Clinical Review Considerations
**Opioids**	Nausea, vomiting, constipation, sedation, and dry mouth	Delirium, falls, fractures, impaired mobility, and reduced ability to self-feed	Confirm ongoing pain indication; optimize nonopioid approaches when appropriate; use the lowest effective dose; prevent and treat constipation; taper rather than abruptly stopping long-term therapy when indicated [46,47].
**Benzodiazepines and nonbenzodiazepine sedative-hypnotics**	Sedation, cognitive impairment, and reduced ability to shop, prepare meals, or self-feed	Delirium, falls, fractures, impaired driving, and functional decline	Avoid when possible; consider nonpharmacologic approaches for insomnia or anxiety and taper gradually when discontinuing to prevent withdrawal [44,46].
**Anticholinergic medications**	Dry mouth, constipation, impaired taste, confusion, and difficulty chewing or swallowing	Cumulative anticholinergic burden, delirium, cognitive decline, falls, and urinary retention	Review all prescription, over the counter, and as-needed products; reduce high-potency anticholinergic exposure when feasible [45,46].
**Antipsychotics and phenothiazines**	Sedation, dry mouth, constipation, swallowing difficulty, and extrapyramidal symptoms	Falls, orthostatic hypotension, delirium, and increased stroke and mortality risk in adults with dementia	Avoid behavioral symptoms of dementia unless nonpharmacologic approaches have failed and there is risk of substantial harm; attempt periodic deprescribing when clinically appropriate [46,47].
**Tricyclic antidepressants**	Dry mouth, constipation, sedation, and appetite or taste changes	Strong anticholinergic effects, orthostatic hypotension, falls, and cognitive impairment	Consider safer alternatives for insomnia, neuropathic pain, or depression while accounting for indication and response [45,46].
**Selective serotonin reuptake inhibitors and serotonin–norepinephrine reuptake inhibitors**	Nausea, appetite reduction, diarrhea, and taste changes	Falls/fractures and syndrome of inappropriate antidiuretic hormone secretion with hyponatremia	Monitor appetite, weight, falls, and serum sodium, particularly after initiation or dose changes in high-risk patients [46,47].
**Cholinesterase inhibitors**	Nausea, vomiting, diarrhea, anorexia, and weight loss	Bradycardia, syncope, falls, dehydration, and functional decline	Monitor weight, oral intake, hydration, heart rate, and gastrointestinal symptoms; periodically reassess cognitive and functional benefit [45,47].
**Digoxin**	Anorexia, nausea, vomiting, and taste changes	Toxicity risk with impaired renal clearance; weakness, delirium, and arrhythmias with toxicity	Monitor kidney function, electrolytes, dose, interacting medications, and symptoms of toxicity; measure drug concentrations when clinically indicated [46,47].
**Amiodarone**	Nausea, anorexia, and altered taste	Thyroid, pulmonary, hepatic, and neurologic toxicities may impair intake and function	Confirm indication and monitor thyroid, liver, pulmonary, neurologic, and gastrointestinal effects; consider alternatives where appropriate [47].
**Diuretics**	Dehydration, electrolyte abnormalities, and possible thiamine loss with chronic loop-diuretic use	Orthostatic hypotension, falls, weakness, renal dysfunction, and gout	Monitor volume status, orthostatic vital signs, electrolytes, kidney function, fluid intake, and weight trends; distinguish fluid loss from loss of lean tissue or fat [45].
**Alpha-1 adrenergic blockers and central alpha-agonists**	Dry mouth, fatigue, and nausea	Orthostatic hypotension, bradycardia, syncope, and falls	Avoid routine use for hypertension in older adults when safer alternatives exist; review indication and individualized blood-pressure goals [46].
**Angiotensin-converting enzyme inhibitors and angiotensin II receptor blockers**	Altered taste in some patients and diet burden from hyperkalemia-related restrictions	Hypotension, hyperkalemia, and renal dysfunction may indirectly impair function and intake	Ask about food avoidance and taste changes; avoid unnecessary dietary restriction; monitor renal function and serum potassium [45].
**Proton-pump inhibitors**	Long-term use may contribute to vitamin B12, magnesium, iron, or calcium absorption concerns in selected patients	*Clostridioides difficile* infection, fracture, and other risks with unnecessary chronic use	Use the lowest effective dose for the shortest appropriate duration; reassess indication periodically and evaluate suspected nutrient deficiencies clinically [45,46].
**Metformin**	Nausea, diarrhea, reduced appetite, and long-term association with vitamin B12 deficiency	Impaired renal function can increase adverse-effect risk; vitamin B12 deficiency may contribute to anemia, neuropathy, and functional decline	Assess gastrointestinal tolerance and weight trend; consider vitamin B12 testing in long-term therapy or with anemia/neuropathy; adjust therapy for renal function [44,45].
**Antibiotics**	Nausea, diarrhea, altered taste, and gut-microbiome disruption	*Clostridioides difficile* infection, dehydration, delirium, and drug interactions	Confirm indication and shortest effective duration; monitor hydration, stool frequency, food intake, renal dosing, and interaction burden [44].
**Laxatives**	Diarrhea, dehydration, and electrolyte losses with overuse	Weakness, orthostatic hypotension, dehydration, and renal dysfunction	Confirm indication and bowel plan; monitor stool frequency, hydration, electrolyte status, and continued need for therapy [44].
**Chemotherapy and targeted cancer therapies**	Nausea, vomiting, mucositis, altered taste, diarrhea, and anorexia	Rapid weight and muscle loss, fatigue, infection risk, and functional decline	Refer early to a registered dietitian; manage symptoms and closely monitor intake, hydration, weight, muscle loss, and treatment tolerance [44].
**Glucagon-like peptide-1 receptor agonists**	Appetite reduction, nausea, vomiting, early satiety, delayed gastric emptying, and weight loss	Dehydration and excessive weight or lean-mass loss in frail or malnourished adults	Assess frailty, nutrition risk, intake, hydration, weight-loss rate, and muscle function before and during therapy; individualize dose and continuation [45].
**Sodium-glucose cotransporter-2 inhibitors**	Weight loss and volume depletion	Orthostatic hypotension, dehydration, genitourinary infections, and potential functional decline in vulnerable adults	Monitor hydration, renal function, orthostatic symptoms, intake, and weight trajectory; direct causation of sarcopenia remains uncertain [45].

Note. This table summarizes potential medication-related contributors to poor nutrition and frailty; it does not imply that medications should be stopped automatically. Review each medication’s indication, benefit, dose, duration, interactions, organ function, treatment goals, and potential alternatives before changing therapy [44,45,46].

## Data Availability

No new data were created or analyzed in this study. Data sharing is not applicable to this article.

## References

[1] Dent E., Morley J.E., Cruz-Jentoft A.J., Woodhouse L., Rodríguez-Mañas L., Fried L.P., Woo J., Aprahamian I., Sanford A., Lundy J. (2019). Physical frailty: ICFSR international clinical practice guidelines for identification and management. J. Nutr. Health Aging.

[2] Fried L.P., Tangen C.M., Walston J., Newman A.B., Hirsch C., Gottdiener J., Seeman T., Tracy R., Kop W.J., Burke G. (2001). Frailty in older adults: Evidence for a phenotype. J. Gerontol. Ser. A.

[3] Millan-Domingo F., Garcia-Dominguez E., Gambini J., Olaso-Gonzalez G., Viña J., Gomez-Cabrera M.C. (2024). Diet and exercise in frailty and sarcopenia. Molecular aspects. Mol. Asp. Med..

[4] Castillo-Angeles M., Cooper Z., Jarman M.P., Sturgeon D., Salim A., Havens J.M. (2021). Association of frailty with morbidity and mortality in emergency general surgery by procedural risk level. JAMA Surg..

[5] Poco L.C., Finkelstein E., Sim D., Malhotra C. (2024). Weak grip strength predicts higher unplanned healthcare utilization among patients with heart failure. ESC Heart Fail..

[6] Verduri A., Carter B., Laraman J., Rice C., Clini E., Maskell N.A., Hewitt J. (2023). Frailty and its influence on mortality and morbidity in COPD: A systematic review and meta-analysis. Intern. Emerg. Med..

[7] Wilson N., Mullaney W. (2024). Frailty and nutrition. Br. J. Community Nurs..

[8] Moon S., Oh E., Chung D., Choi R., Hong G.-R.S. (2023). Malnutrition as a major related factor of frailty among older adults residing in long-term care facilities in Korea. PLoS ONE.

[9] Hewitt J., Carter B., McCarthy K., Pearce L., Law J., Wilson F.V., Tay H.S., McCormack C., Stechman M.J., Moug S.J. (2019). Frailty predicts mortality in all emergency surgical admissions regardless of age: An observational study. Age Ageing.

[10] Gracie T.J., Caufield-Noll C., Wang N.Y., Sieber F.E. (2021). The association of preoperative frailty and postoperative delirium: A meta-analysis. Anesth. Analg..

[11] Zhang Y., Wu Q., Han M., Yang C., Kang F., Li J., Hu C., Chen X. (2024). Frailty is a risk factor for postoperative complications in older adults with lumbar degenerative disease: A prospective cohort study. Clin. Interv. Aging.

[12] Shah R., Borrebach J.D., Hodges J.C., Varley P.R., Wisniewski M.K., Shinall M.C., Arya S., Johnson J., Nelson J.B., Youk A. (2020). Validation of the Risk Analysis Index for evaluating frailty in ambulatory patients. J. Am. Geriatr. Soc..

[13] Dicpinigaitis A.J., Khamzina Y., Hall D.E., Nassereldine H., Kennedy J., Seymour C.W., Schmidt M., Reitz K.M., Bowers C.A. (2024). Adaptation of the Risk Analysis Index for frailty assessment using diagnostic codes. JAMA Netw. Open.

[14] Hall D.E., Arya S., Schmid K.K., Blaser C., Carlson M.A., Bailey T.L., Purviance G., Bockman T., Lynch T.G., Johanning J. (2017). Development and initial validation of the Risk Analysis Index for measuring frailty in surgical populations. JAMA Surg..

[15] Hall D.E., Arya S., Schmid K.K., Carlson M.A., Lavedan P., Bailey T.L., Purviance G., Bockman T., Lynch T.G., Johanning J.M. (2017). Association of a frailty screening initiative with postoperative survival at 30, 180, and 365 days. JAMA Surg..

[16] Hall D.E., Hagan D., Ashcraft L.E., Wilson M., Arya S., Johanning J.M. (2025). The Surgical Pause: The importance of measuring frailty and taking action to address identified frailty. Jt. Comm. J. Qual. Patient Saf..

[17] Arya S., Varley P., Youk A., Borrebach J.D., Perez S., Massarweh N.N., Johanning J.M., Hall D.E. (2020). Recalibration and external validation of the Risk Analysis Index: A surgical frailty assessment tool. Ann. Surg..

[18] Moon H., Lee G.H., Kim D.H. (2024). Frailty: Assessment and intervention. Keimyung Med. J..

[19] Ng Y.X., Cheng L.J., Quek Y.Y., Yu R., Wu X.V. (2024). The measurement properties and feasibility of FRAIL scale in older adults: A systematic review and meta-analysis. Ageing Res. Rev..

[20] Lee H., Lee E., Jang I.Y. (2020). Frailty and comprehensive geriatric assessment. J. Korean Med. Sci..

[21] Briggs R., McDonough A., Ellis G., Bennett K., O’Neill D., Robinson D. (2022). Comprehensive geriatric assessment for community-dwelling, high-risk, frail, older people. Cochrane Database Syst. Rev..

[22] Church S., Rogers E., Rockwood K., Theou O. (2020). A scoping review of the Clinical Frailty Scale. BMC Geriatr..

[23] Rockwood K., Song X., MacKnight C., Bergman H., Hogan D.B., McDowell I., Mitnitski A. (2005). A global clinical measure of fitness and frailty in elderly people. CMAJ.

[24] Mehmet H., Yang A.W.H., Robinson S.R. (2020). Measurement of hand grip strength in the elderly: A scoping review with recommendations. J. Bodyw. Mov. Ther..

[25] Mehmet H., Yang A.W.H., Robinson S.R. (2020). What is the optimal chair stand test protocol for older adults? A systematic review. Disabil. Rehabil..

[26] de Souza A.F., de Oliveira D.C., Ramírez P.C., de Oliveira Máximo R., Luiz M.M., Delinocente M.L.B., Steptoe A., de Oliveira C., da Silva Alexandre T. (2025). Low gait speed is better than frailty and sarcopenia at identifying the risk of disability in older adults. Age Ageing.

[27] Centers for Disease Control and Prevention (2017). Timed Up & Go (TUG) [Assessment Tool]. https://www.cdc.gov/steadi/media/pdfs/STEADI-Assessment-TUG-508.pdf.

[28] Guralnik J.M., Simonsick E.M., Ferrucci L., Glynn R.J., Berkman L.F., Blazer D.G., Scherr P.A., Wallace R.B. (1994). A short physical performance battery assessing lower extremity function: Association with self-reported disability and prediction of mortality and nursing home admission. J. Gerontol..

[29] Yang H., Wang B., Wang Q., Zhao J., Liu F., Xie X., Xu F., Zhang P. (2026). Effect of multicomponent exercise and nutrition intervention on frailty status in older adults: A network meta-analysis. BMC Geriatr..

[30] Volkert D., Beck A.M., Cederholm T., Cruz-Jentoft A., Hooper L., Kiesswetter E., Maggio M., Raynaud-Simon A., Sieber C., Sobotka L. (2022). ESPEN practical guideline: Clinical nutrition and hydration in geriatrics. Clin. Nutr..

[31] Cruz-Jentoft A.J., Volkert D. (2025). Malnutrition in older adults. N. Engl. J. Med..

[32] Cederholm T., Jensen G.L., Correia M.I.T.D., Gonzalez M.C., Fukushima R., Higashiguchi T., Baptista G., Barazzoni R., Blaauw R., Coats A.J.S. (2019). GLIM criteria for the diagnosis of malnutrition: A consensus report from the global clinical nutrition community. J. Cachexia Sarcopenia Muscle.

[33] Serón-Arbeloa C., Labarta-Monzón L., Puzo-Foncillas J., Mallor-Bonet T., Lafita-López A., Bueno-Vidales N., Montoro-Huguet M. (2022). Malnutrition screening and assessment. Nutrients.

[34] Le Thi Thanh X., Duong Thi P., Le Thi H., Phung Lam T., Nguyen Quang D., Nguyen Thi Huong L., Luu Thi My T., Nguyen Thi Thuy H. (2025). Validity of NRS-2002, MUST, MST, and MNA-SF as first-step screening tools for malnutrition based on GLIM criteria in older adults. Clin. Nutr. Open Sci..

[35] Kaiser M.J., Bauer J.M., Ramsch C., Uter W., Guigoz Y., Cederholm T., Thomas D.R., Anthony P., Charlton K.E., Maggio M. (2009). Validation of the Mini Nutritional Assessment short-form (MNA-SF): A practical tool for identification of nutritional status. J. Nutr. Health Aging.

[36] Ferguson M., Capra S., Bauer J., Banks M. (1999). Development of a valid and reliable malnutrition screening tool for adult acute hospital patients. Nutrition.

[37] White J.V., Guenter P., Jensen G., Malone A., Schofield M., Academy Malnutrition Work Group, A.S.P.E.N. Malnutrition Task Force, A.S.P.E.N. Board of Directors (2012). Consensus statement: Academy of Nutrition and Dietetics and American Society for Parenteral and Enteral Nutrition: Characteristics recommended for the identification and documentation of adult malnutrition (undernutrition). J. Parenter. Enter. Nutr..

[38] Jensen G.L., Cederholm T., Correia M.I.T.D., Gonzalez M.C., Fukushima R., Pisprasert V., Blaauw R., Braz D.C., Carrasco F., Cruz Jentoft A.J. (2025). GLIM consensus approach to diagnosis of malnutrition: A 5-year update. J. Parenter. Enter. Nutr..

[39] Xu Y., Ji T., Li X., Yang Y., Zheng L., Qiu Y., Chen L., Li G. (2024). The effectiveness of the comprehensive geriatric assessment for older adults with frailty in hospital settings: A systematic review and meta-analysis. Int. J. Nurs. Stud..

[40] Tan X., Miu X., Wang X., Li X., Chen X., Wen X. (2025). Comprehensive geriatric assessment for older adults admitted to the hospital as emergencies: A meta-analysis of randomized controlled trials. Geriatr. Nurs..

[41] Kumlehn B., Brefka S., Kocar T., Verri F.M., Wirth R., Denkinger M. (2025). Clinical practice guideline: Comprehensive geriatric assessment in the hospital. Dtsch. Ärzteblatt Int..

[42] Vendola M.C.C., Jacob-Filho W. (2023). Impact of oral health on frailty syndrome in frail older adults. Einstein.

[43] Dibello V., Zupo R., Sardone R., Lozupone M., Castellana F., Dibello A., Daniele A., De Pergola G., Bortone I., Lampignano L. (2021). Oral frailty and its determinants in older age: A systematic review. Lancet Healthy Longev..

[44] Kose E., Wakabayashi H., Yasuno N. (2021). Polypharmacy and malnutrition management of elderly perioperative patients with cancer: A systematic review. Nutrients.

[45] Norman K., Haß U., Pirlich M. (2021). Malnutrition in older adults—Recent advances and remaining challenges. Nutrients.

[46] (2023). American Geriatrics Society Beers Criteria^®^ Update Expert Panel. American Geriatrics Society 2023 updated AGS Beers Criteria^®^ for potentially inappropriate medication use in older adults. J. Am. Geriatr. Soc..

[47] Hutchison L.C. (2022). Management of Weight Loss in Hospitalized Older Adults. University of Arkansas for Medical Sciences, Arkansas Geriatric Education Collaborative. https://agec.uams.edu/weightlossmgmt/.

[48] Harris S., DePalma J., Barkoukis H. (2025). Protein and aging: Practicalities and practice. Nutrients.

[49] Gillis C., Ljungqvist O., Carli F. (2022). Prehabilitation, enhanced recovery after surgery, or both? A narrative review. Br. J. Anaesth..

[50] Park Y., Choi J.E., Hwang H.S. (2018). Protein supplementation improves muscle mass and physical performance in undernourished prefrail and frail elderly subjects: A randomized, double-blind, placebo-controlled trial. Am. J. Clin. Nutr..

[51] Kirwan R.P., Mazidi M., Rodríguez García C., Lane K.E., Jafari A., Butler T., Perez de Heredia F., Davies I.G. (2022). Protein interventions augment the effect of resistance exercise on appendicular lean mass and handgrip strength in older adults: A systematic review and meta-analysis of randomized controlled trials. Am. J. Clin. Nutr..

[52] Xue Q., Shen M., Lin Q., Wu X., Yang M. (2023). The association between dietary protein diversity and protein patterns with frailty in older Chinese adults: A population-based cohort study. J. Nutr. Health Aging.

[53] Bauer J., Biolo G., Cederholm T., Cesari M., Cruz-Jentoft A.J., Morley J.E., Phillips S., Sieber C., Stehle P., Teta D. (2013). Evidence-based recommendations for optimal dietary protein intake in older people: A position paper from the PROT-AGE Study Group. J. Am. Med. Dir. Assoc..

[54] Poursalehi D., Lotfi K., Saneei P. (2023). Adherence to the Mediterranean diet and risk of frailty and pre-frailty in elderly adults: A systematic review and dose-response meta-analysis with GRADE assessment. Ageing Res. Rev..

[55] Gross D.C., Dahringer J.C., Bramblett P., Sun C., Spangler H.B., Lynch D.H., Batsis J.A. (2025). The relationship between a Mediterranean diet and frailty in older adults: NHANES 2007–2017. Nutrients.

[56] Kojima G., Avgerinou C., Iliffe S., Walters K. (2018). Adherence to Mediterranean diet reduces incident frailty risk: Systematic review and meta-analysis. J. Am. Geriatr. Soc..

[57] Donat-Vargas C., Domínguez L.J., Sandoval-Insausti H., Moreno-Franco B., Rey-Garcia J., Banegas J.R., Rodríguez-Artalejo F., Guallar-Castillón P. (2022). Olive oil consumption is associated with lower frailty risk: A prospective cohort study of community-dwelling older adults. Age Ageing.

[58] Kim J., Westra J., Tintle N., Harris W.S., Park Y. (2024). Association of plasma n-3 polyunsaturated fatty acid levels and the prevalence of frailty in older adults: A cross-sectional analysis of UK Biobank. J. Gerontol. Ser. A.

[59] Urpi-Sarda M., Andres-Lacueva C., Rabassa M., Ruggiero C., Zamora-Ros R., Bandinelli S., Ferrucci L., Cherubini A. (2015). The relationship between urinary total polyphenols and the frailty phenotype in a community-dwelling older population: The InCHIANTI study. J. Gerontol. Ser. A.

[60] Wu M., Luo Q., Nie R., Yang X., Tang Z., Chen H. (2021). Potential implications of polyphenols on aging considering oxidative stress, inflammation, autophagy, and gut microbiota. Crit. Rev. Food Sci. Nutr..

[61] Wen N.N., Sun L.W., Geng Q., Zheng G.H. (2024). Gut microbiota changes associated with frailty in older adults: A systematic review of observational studies. World J. Clin. Cases.

[62] Ghosh T.S., Rampelli S., Jeffery I.B., Santoro A., Neto M., Capri M., Giampieri E., Jennings A., Candela M., Turroni S. (2020). Mediterranean diet intervention alters the gut microbiome in older people reducing frailty and improving health status: The NU-AGE 1-year dietary intervention across five European countries. Gut.

[63] Wobith M., Strobel R., Klek S., Ljungqvist O., Wischmeyer P., Weimann A. (2026). Perioperative nutrition therapy in high-risk patients: A narrative review. Nutr. Clin. Pract..

[64] Abizanda Saro A., García Molina R., Alcantud Córcoles R., Maestre Moreno M., Sánchez Uceda R., Morón Merchante I., Aragonés Jiménez A., González Céspedes M.D., Montero Alía P., Simarro Rueda M. (2026). Efficacy of a Multicomponent Intervention for Frailty or Physical Function in Prefrail or Frail Older Adults: FRAILMERIT Multicenter Clinical Trial. J. Am. Geriatr. Soc..

[65] Del Moral-Pairada M., Laserna Jiménez C., Fabrellas N., Lopez-Poyato M. (2025). Exploring nursing interventions for frail individuals in primary care: A systematic review. Int. Nurs. Rev..

[66] Morón-Duarte L.S., Chacón-Acevedo K.R., Ossa W.D., Gomez Gomez O.V., Stampini M., Aranco N., Yomayusa-González N. (2026). Effectiveness of social and community health interventions in older adults with frailty, pre-frailty, or at risk of frailty: An overview of systematic reviews. Arch. Gerontol. Geriatr. Plus.

[67] Cacchione P.Z. (2020). Age-friendly health systems: The 4Ms framework. Clin. Nurs. Res..

[68] U.S. Department of Veterans Affairs The Surgical Pause. VA Diffusion Marketplace. 10 January 2024. https://marketplace.va.gov/innovations/preoperative-frailty-screening-prehabilitation.

[69] Varley P.R., Buchanan D., Bilderback A., Wisniewski M.K., Johanning J., Nelson J.B., Johnson J.T., Minnier T., Hall D.E. (2023). Association of routine preoperative frailty assessment with 1-year postoperative mortality. JAMA Surg..

[70] Sadlonova M., Katz N.B., Jurayj J.S., Flores L., Celano C.M., von Arnim C.A.F., Silver J.K. (2023). Surgical prehabilitation in older and frail individuals: A scoping review. Int. Anesthesiol. Clin..

[71] Molenaar C.J.L., Minnella E.M., Coca-Martinez M., Ten Cate D.W.G., Regis M., Awasthi R., Martínez-Palli G., López-Baamonde M., Sebio-Garcia R., Feo C.V. (2023). Effect of multimodal prehabilitation on reducing postoperative complications and enhancing functional capacity following colorectal cancer surgery: The PREHAB randomized clinical trial. JAMA Surg..

